# Effect of Antigen Shedding on Targeted Delivery of Immunotoxins in Solid Tumors from a Mathematical Model

**DOI:** 10.1371/journal.pone.0110716

**Published:** 2014-10-24

**Authors:** Youngshang Pak, Ira Pastan, Robert J. Kreitman, Byungkook Lee

**Affiliations:** 1 Department of Chemistry and Institute of Functional Materials, Pusan National University, Busan, Republic of Korea; 2 Laboratory of Molecular Biology, National Cancer Institute, National Institutes of Health, Bethesda, Maryland, United States of America; University of Glasgow, United Kingdom

## Abstract

Most cancer-specific antigens used as targets of antibody-drug conjugates and immunotoxins are shed from the cell surface (Zhang & Pastan (2008) Clin. Cancer Res. 14: 7981-7986), although at widely varying rates and by different mechanisms (Dello Sbarba & Rovida (2002) Biol. Chem. 383: 69–83). Why many cancer-specific antigens are shed and how the shedding affects delivery efficiency of antibody-based protein drugs are poorly understood questions at present. Before a detailed numerical study, it was assumed that antigen shedding would reduce the efficacy of antibody-drug conjugates and immunotoxins. However, our previous study using a comprehensive mathematical model showed that antigen shedding can significantly improve the efficacy of the mesothelin-binding immunotoxin, SS1P (anti-mesothelin-Fv-PE38), and suggested that receptor shedding can be a general mechanism for enhancing the effect of inter-cellular signaling molecules. Here, we improved this model and applied it to both SS1P and another recombinant immunotoxin, LMB-2, which targets CD25. We show that the effect of antigen shedding is influenced by a number of factors including the number of antigen molecules on the cell surface and the endocytosis rate. The high shedding rate of mesothelin is beneficial for SS1P, for which the antigen is large in number and endocytosed rapidly. On the other hand, the slow shedding of CD25 is beneficial for LMB-2, for which the antigen is small in number and endocytosed slowly.

## Introduction

Recombinant immunotoxins (immunotoxins for short) and antibody-drug conjugates (ADCs) are protein and chemical toxins, respectively, that are conjugated to an antibody or antibody fragment. As promising anti-cancer agents, immunotoxins and antibody-drug conjugates are designed to kill only the cancer cells by binding to specific target antigens expressed on tumor cell surface [1]–[3]. In this report, we consider the delivery efficiency of immunotoxins only, although a similar consideration will apply for the antibody-drug conjugates as well.

**Figure 1:**
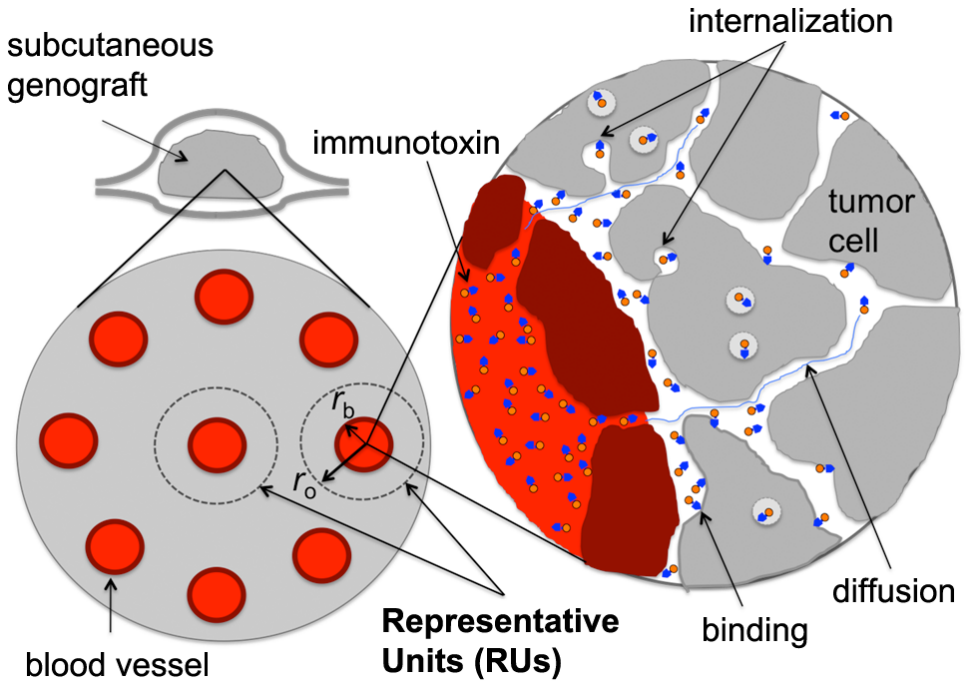
The vascular tumor model. It consists of a collection of representative units (RUs), each a cylinder of radius *r_0_* centered on a cylindrical blood vessel of radius *r_b_*, which serves as the source of the administered immunotoxin. The values of *r_b_* and *r_0_* used were 11 and 50 µm, respectively. Expanded view: The immunotoxin molecules, each represented as a small orange circle with a blue arrowhead, leave from blood vessel by permeating through the blood vessel wall into the extra-cellular space of the tumor tissue, diffuse in this space, bind to surface antigen molecules, and then become internalized by endocytosis.

The potency of an immunotoxin can be reduced by incomplete penetration through the solid tumor tissue [4]. The immunotoxin delivery process to solid tumors consists of a series of kinetic events. Upon injection into the blood stream, immunotoxin molecules permeate through the blood vessel wall into the extra-cellular space of the tumor, diffuse in the extra-cellular space, and bind to and dissociate from surface antigen molecules on tumor cells (Fig. 1). The surface bound immunotoxin molecules are internalized by receptor-mediated endocytosis and processed. The processed toxin molecules are then translocated into the cytosol where they begin the process that leads to cell death. Understanding these long and complex kinetic events for the immunotoxin delivery process is of practical importance for designing a better delivery strategy to improve the efficacy of immunotoxins.

In preclinical tests, different doses of immunotoxin are injected into the blood stream of tumor-bearing mice and subsequently tumor volume changes are monitored with time. These dose-dependent anti-tumor activity data, along with *in vitro* binding and cytotoxicity data, give a valuable insight into the factors that determine the effectiveness of immunotoxins. We previously reported on a mathematical model [5], which consisted of a system of partial differential equations and incorporated many key experimentally determined biological variables. It tracks the concentration of immunotoxin in all parts of the tumor tissue at all times. This model was applied to SS1P (anti-mesothelin-Fv-PE38), which is an immunotoxin targeted to mesothelin, a protein antigen highly expressed in several cancers [6]. SS1P is an excellent benchmark system for the development of our mathematical model because extensive pharmacokinetic parameters are available for this system from careful preclinical studies [7], [8]. The model reproduced experimental tumor response data upon injection of different amounts of SS1P. It also exhibited the well-known binding site barrier effect [4], [9], which refers to the hindrance to penetration of the antibody-based toxin into the tumor tissue by excessive binding to antigens on the cells nearby the blood vessel.

The most interesting finding was related to the effect of antigen shedding. Most cancer-specific antigens are shed into the extracellular space and into the blood [10], [11]. Prior to this study, the antigen shedding was expected to hinder the immunotoxin delivery [11] since the shed antigen can act as a decoy for the immunotoxins and since the immunotoxin-antigen complex formed on the cell surface can be shed before being internalized. However, the model unexpectedly showed that receptor shedding enhanced the delivery efficacy of SS1P in solid tumors [5]. This was because shed antigen molecules in the extra-cellular space became a reservoir and carrier of immunotoxins and promoted a more uniform immunotoxin distribution in the tumor tissue by circumventing the binding site barrier effect. Based on this new finding, several suggestions were made to improve the effectiveness of immunotoxins in treating solid tumors.

Here we present a new, improved model. The modifications made are described in the Methods section and in more detail in Text S1. They are mostly technical and, with a couple of exceptions, result in small, although clearly discernable, improvements in the fit between the calculated and experimentally measured changes in volume of human tumors growing in mice with time after immunotoxin administration. Briefly, the two important exceptions are: (1) Back permeation into blood is allowed for all species including the shed antigen-immunotoxin complex. Previous model allowed back permeation of only the shed antigen species. This change results in a marked reduction of the beneficial effect of shed antigen. And (2) the permeation rate constant is reduced when the tumor size becomes large. This is not so much to reflect that the permeability of the capillaries in tumor varies with tumor size, although it may, but to effectively mimic the condition of very aberrant and relatively poorer vascularization for larger tumors [12], [13]. In the previous model, the permeation rate constant was kept fixed, independent of tumor size. This change is needed in order to reproduce the experimental observation that the shed antigen level in the extracellular space of solid tumors is larger for larger tumors [8].

We applied the new model to SS1P and another immunotoxin, LMB-2. LMB-2 (anti-Tac-Fv-PE38) is an anti-CD25 immunotoxin targeting CD25 displayed on cells of various hematological malignancies [14]. LMB-2 and SS1P share the same toxin fragment of Pseudomonas exotoxin A (PE). The preclinical efficacy data for SS1P were obtained using mice that bear the mesothelin expressing A431/H9 cell derived tumors. Similar data exist for LMB-2 using mice that bear ATAC-4 cell derived solid tumors [15]. A431/H9 and ATAC-4 cells are both A431 cells (human epidermoid carcinoma cell line), the former transfected with mesothelin, and the latter with CD25, expressing vectors. Thus, the two systems are very similar. But they also have important differences. For example, the number of antigen molecules on the surface is measured to be ∼10^6^ per cell in the case of the mesothelin expressing A431/H9 cells [7] whereas it is only about 2×10^5^ for the CD25 expressing ATAC-4 cells [16]. Both antigens are membrane-bound, mesothelin by a GPI anchor [17] and CD25 by a trans-membrane peptide segment near the C-terminus [18]. Both are shed from the cell surface apparently by proteolytic cleavage from the membrane-bound C-terminal region of the polypeptide chain [19], [20]. But the shedding rate is estimated to be about 20% per hour for mesothelin and only 0.2% per hour for CD25, according to our analysis (see Methods) of the experimental data [7], [21]. Such similarities and differences, as well as the availability of extensive data, make these two systems highly valuable for testing and refining our mathematical model. The model includes some 31 parameters (Table 1), 18 of which have the same values for both systems (Table 2) while 13 others have different values (Table 3).

**Table 1:** Symbols, definitions, and units of the model parameters. (ECS: extra-cellular space).

Parameters	Unit	Definition
*Mw (immunotoxin)*	kDa	Molecular weight of immunotoxin
*Mw (shed antigen)*	kDa	Molecular weight of shed-antigen
R*	number/cell	Number of total surface receptors per cell
ρ*	cells/cm^3^	Tumor cell density in EVS
*φ_e_*		Volume fraction of the tumor extracellular space per tumor extravascular volume
*r_b_*	_µm_	Blood vessel radius
*r_o_*	_µm_	Outer radius of RU
*t_α_ (immunotoxin)*	min.	Half-life of immunotoxin in the blood
*t_α_ (shed antigen)*	min.	Half-life of shed-antigen in the blood
*P^f^_efT_ (low)*	cm/s	Lower value of forward permeability (to ECS)
*P^f^_efT_ (high)*	cm/s	Higher value of forward permeability
*V_c_*	_mm_^3^	Volume at which the permeability is the average of the low and high values.
*a*	_mm_^−3^	Slope of the sigmoidal function for permeability at *V_c_*
*D_efT_*	cm^2^/s	Diffusion constant of immunotoxin in ECS
*χ_efT_*	hr^−1^	Degradation rate constant of immunotoxin in ECS
*χ_efR_*	hr^−1^	Degradation rate constant of free antigen in ECS
*χ_ecR_*	hr^−1^	Degradation rate constant of complexed antigen in ECS
*χ_ce_*	hr^−1^	Endosomal degradation rate constant of immunotoxin
*χ_cc_*	hr^−1^	Cytosolic degradation rate constant of immunotoxin
*k_d_*	hr^−1^	Immunotoxin-receptor dissociation rate constant
*k_a_*	hr^−1^ nM^−1^	Immunotoxin-receptor association rate constant
*K_D_≡ k_d_/k_a_*	nM	Binding affinity of immunotoxins to receptors
*k_s_*	hr^−1^	Receptor shedding rate constant
*k_e_*	hr^−1^	Endocytosis rate constant
*k_t_*	hr^−1^	Immunotoxin translocation rate constant
*Ω≡ k_t_/(χ_ce_+k_t_)*		Fraction translocated from endosome
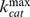	hr^−1^	Maximum intoxication rate constant
*T_0_*		Number of cytosolic toxin molecules per type 1 cell at which the intoxication rate is half of 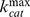
*Γ_0_*	hr^−1^	Tumor growth rate constant at small volume
*α*	_mm_^−3^	Tumor growth rate decay parameter with tumor volume
*Δ*	hr^−1^	Cell death rate constant
*Χ*	Hr^−1^	Dead cell clearance rate constant

**Table 2:** Parameters that were unchanged between SS1P and LMB-2.

Parameters	SS1P LMB-2	Comment/Reference
ρ* (cells/cm^3^)	0.5×10^9^	[25]
*φ_e_*	0.1	
*r_b_ (µm)*	11.0	
*r_o_ (µm)*	50.0	Assume
*P^f^_efT_ (low) (cm/s)*	7.1×10^−7^	
*P^f^_efT_ (high)(cm/s)*	8.0×10^−6^	
*V_c_ (mm^3^)*	250.0	
*a (mm^−3^)*	0.04	
*D_efT_ (cm^2^/s)*	2.5×10^−8^	[33]
*χ_efT_ (hr^−1^)*	0.1	
*χ_efR_ (hr^−1^)*	0.1	
*χ_ecR_ (hr^−1^)*	0.1	
*χ_ce_ (hr^−1^)*	0.021	[33]
*k_t_ (hr^−1^)*	0.69	[5]
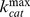 *(hr^−1^)*	0.252	
*T_0_*	400	
*Δ (hr^−1^)*	0.17	
*Χ (hr^−1^)*	0.23	

**Table 3:** Parameters that were changed for LMB-2.

Parameters	SS1P	Comment/Reference	LMB-2	Comment/Reference
*Mw (immunotoxin) (kDa)*	64	[7]	63	[14]
*Mw (shed antigen) (kDa)*	42	[7]	45	[34]
R*	1.0×10^6^	[7]	2.0×10^5^	[16]
*t_α_ (immunotoxin) (min)*	24	[35]	13	
*t_α_ (shed antigen) (min)*	3	[5]	5	
*k_d_ (hr^−1^)*	0.61	u	0.71	Assume
*K_D_ (nM)*	1.2	u	1.4	[22]
*k_s_ (hr^−1^)*	0.2		0.0020	
*k_e_ (hr^−1^)*	0.22		0.08	
*Ω*	0.065		0.012	
*Γ_0_ (hr^−1^)*	0.0301		0.0170	
*α (mm^−3^)*	0.00127		0.00197	
*χ_ce_ (hr^−1^)*	9.9		56.8	

u unpublished data.

SS1P and LMB-2 have comparable affinities to their respective antigens; K_D_ values are 1.2 nM for SS1P (unpublished data) and 1.4 nM for LMB-2 [22]. However, the potencies of the two immunotoxins may well differ since there are more mesothelin molecules on the surface, which are also shed more. Experimental data that directly and clearly compare the efficacies of the two immunotoxins do not exist. However, data from independent experiments suggest that LMB-2 is equally, or somewhat more, effective than SS1P: injection of three 160 µg/kg doses of LMB-2 to ATAC-4 tumor bearing mice [15] appeared to produce more tumor growth suppression than injection of three 200 µg/kg doses of SS1P to A431/H9 tumor bearing mice [7].

Our new model reproduces tumor volume changes for both of these systems. The model shows that shedding can reduce, as well as enhance, antitumor activity depending on the number of antigen molecules on the cell surface: It predicts that shedding is beneficial for SS1P but reduces the efficacy of LMB-2. The fact that antigen shedding can both enhance and retard the efficacy of immunotoxins raises the possibility of a new mechanism by which receptor shedding can regulate signaling in normal tissues [23], [24].

## Results

### Shed antigen levels

**Figure 2:**
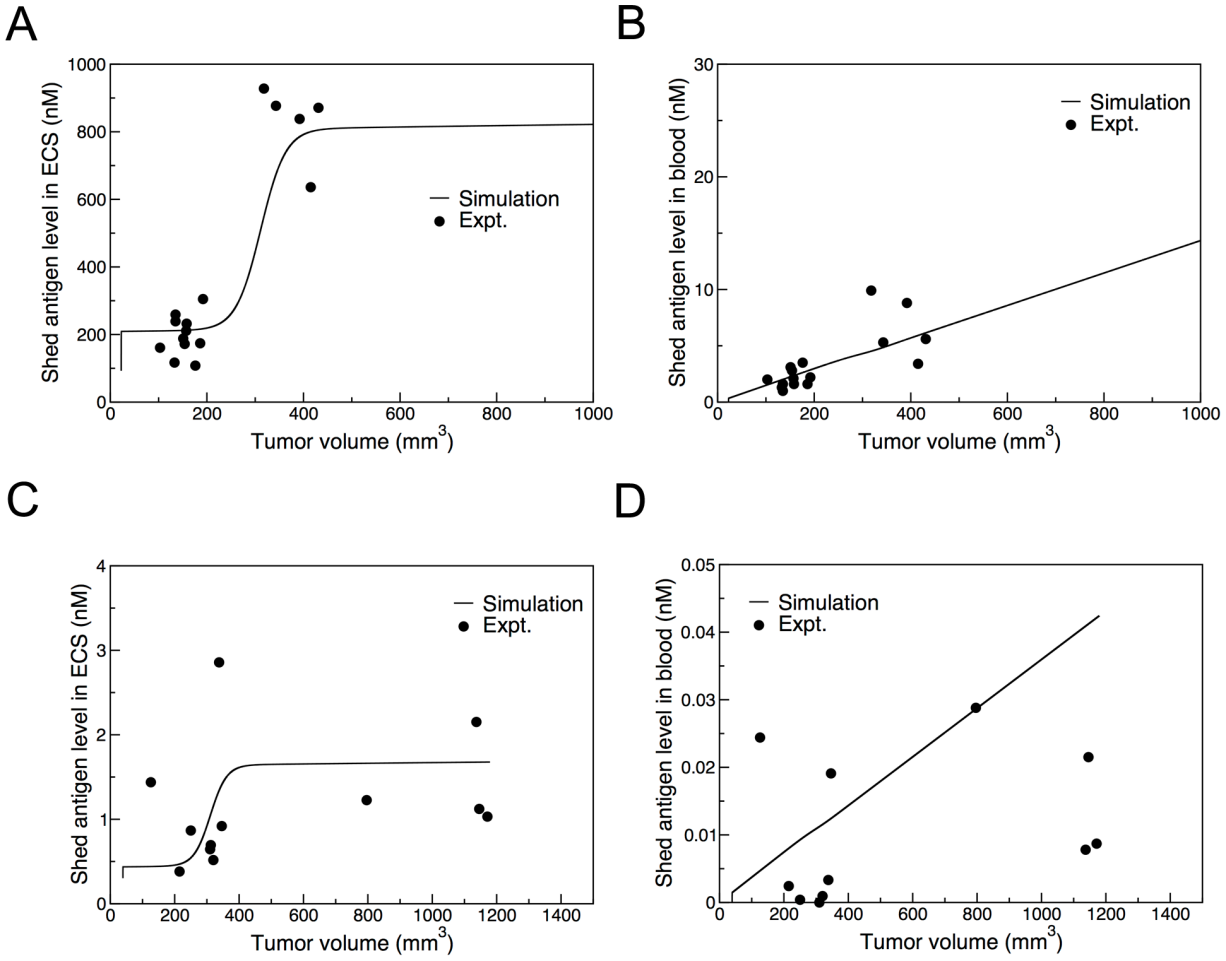
Tumor volume dependence of the measured (symbols) and computed (line) shed antigen levels in the extra-cellular space (A, C) and blood (B, D) for A431/H9 (A, B) and ATAC-4 cells (C, D). The experimental data for mesothelin on A431/H9 cell (panels A and B) are from Zhang et al. [7] and those for CD25 on ATAC-4 cell (panels C and D) are from Singh et al. [15].

Figs. 2A and 2B show the calculated shed antigen levels in the extra-cellular space and blood, respectively, for the mesothelin-expressing A431/H9 tumors without the administration of SS1P. The calculated values reproduce the characteristic feature of the experimental data by Zhang et al. [8] that the shed antigen concentrations in the extra-cellular space and blood plasma both increase with tumor size. The tumor size-dependence of the shed antigen level is produced by the implementation of a new permeability function that reduces the permeability as the tumor volume increases according to Eqs. (2) and (3) in Methods. The previous model used a constant permeability, which led to a flat extra-cellular level with tumor volume.

This tumor cell line expresses a large number of mesothelin molecules (10^6^/cell). The shedding rate constant determined from the best fit of the calculated extra-cellular and blood concentrations of shed antigen to the experimental data is 0.20 hr^−1^ (Table 3). This shedding rate is smaller than the previous value of 0.4 hr^−1^ [5], which was based on *in vitro* experimental data.

Figs. 2C and 2D show the shed antigen (CD25) concentrations for the ATAC-4 tumor. Unlike the case of mesothelin, the shed CD25 concentrations in the extra-cellular space and blood are orders of magnitude smaller and the data points are scattered [15]. Nevertheless, these data provide useful information on the shedding rate: Assuming the same permeability as for the A431/H9 tumor (Table 2), the experimental shed antigen levels can be obtained only when the shedding rate is much smaller than that of mesothelin. Numerically, the shedding rate constant of 0.002 hr^−1^ gives the best fit for CD25.

### Tumor volume responses

**Figure 3:**
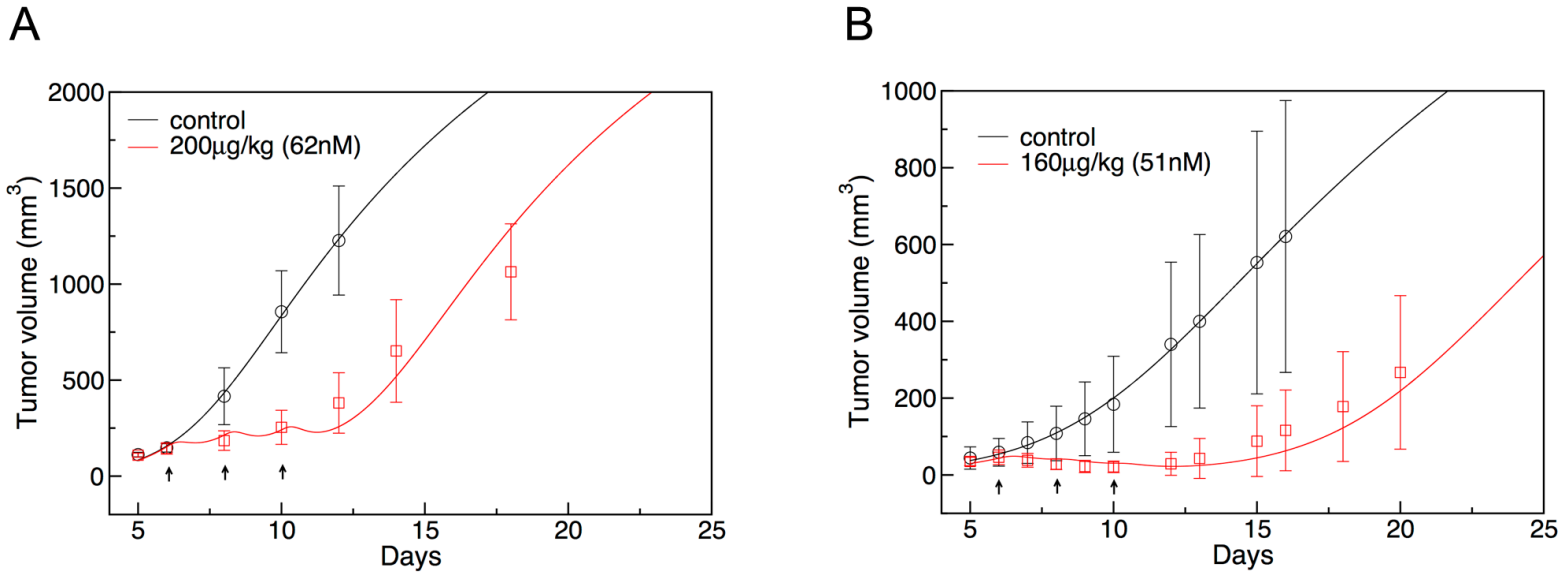
Measured (symbols with error bars) and computed (line) tumor volumes with time of A431/H9 (A) and ATAC-4 (B) cell tumors growing in nude mice. The experimental data are from Zhang et al. [7] and Singh et al. [15]. In each panel, the black line and symbols are for control with no immunotoxin and the red line and symbols are for the case when immunotoxin was given three times, at times indicated by the black arrows. The dose level was 200 µg/kg (62 nM) for SS1P (panel A) and 160 µg/kg (51 nM) for LMB-2 (panel B).

Fig. 3A shows the predicted tumor volume responses upon three SS1P injections with a dose of 200 µg/kg (62 nM) every other day. All calculated volumes fall within the variation of experimental data points. The fit is better than in the previous work on the same system [5], particularly for early and late tumor volumes. The better fit for the early times occurs mainly upon the use of tumor size dependent vascular permeability. The better fit for the late tumor volume data is due to the use of the Gompertzian cell growth model (see Eq. (4) in Methods).

Fig. 3B shows the experimental and simulated tumor volume profiles upon administration of LMB-2 to the ATAC-4 tumor bearing mice. Here, the LMB-2 with a dose of 160 µg/kg (51 nM) was injected three times every other day. The calculated volumes compare well with the recent experimental data of Singh et al. [15].

### Antigen shedding improves the efficacy of SS1P on the A431/H9 tumor

**Figure 4:**
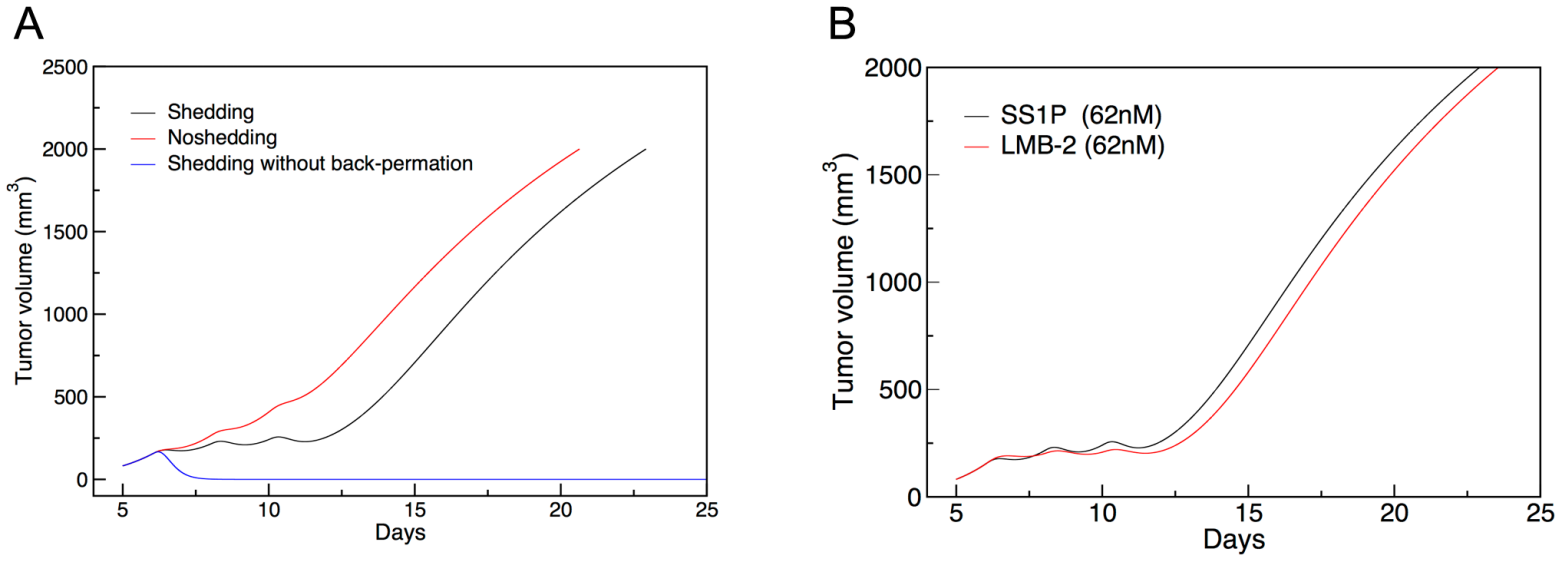
Computed tumor volume profiles using altered model parameters. (**A**) A431/H9 cell tumor with 3×62 nM injections of SS1P for three cases: No shedding (red line) and shedding with (black line; current model) and without (blue line; previous model) the back-permeation of shed antigen-immunotoxin complex to the blood vessel. (**B**) Comparison of the A431/H9 cell tumor with SS1P injection (black line) and ATAC-4 cell tumor with LMB-2 injection (red line) using the same dose (62 nM), initial volume (110 mm^3^), and tumor growth rate (*Γ_0_* = 0.0301 hr^−1^, *α* = 0.00127 mm^−3^).

The shed antigen is found in the blood as well as in the extra-cellular space [7], [11]. In this work, we assume that both the shed antigen and the shed antigen-immunotoxin complex that forms in the extra-cellular space back-permeate to the blood. We investigated the effect of the back-permeation on the tumor volume response upon the administration of SS1P. Fig. 4A shows the simulated tumor volume profiles for the non-shedding case (red line) and the shedding case with (black line) and without (blue line) back-permeation of the shed antigen-immunotoxin complex. The latter case corresponds to our previous model wherein the back-permeation rate for the antigen-immunotoxin complex was set to zero. In the current model, the back-permeation is allowed for the complex with the permeability set to be the same as that for the shed antigen and the same as the forward (from blood to the tumor extra-cellular space) permeability (see Methods). The Figure shows that antigen shedding improves the efficacy of SS1P in both models. However, the improvement is much less when back-permeation is allowed (current model) than when it is not allowed (previous model). Thus, the beneficial effect of antigen shedding reported in the previous study may have been over-estimated (see Discussion).

### SS1P and LMB-2 have a similar predicted potency

Having demonstrated the robust fitting capability with our refined models, next we compared the *in vivo* potency of these two immunotoxins. In order to make the comparison simple, we made the initial conditions for tumor volume simulations the same by keeping their initial tumor volume to 110 mm^3^ for both tumors and the dose to 62 nM for both immunotoxins. As seen from the tumor growth profiles without toxins (the control curves in Fig. 3), the experimental tumor growth rates for the A431/H9 and ATAC-4 cells are quite different, although both are derived from the same cell line. For the efficacy test, any bias resulting from two different tumor cell growth rates needs to be eliminated. For the purpose of comparison, we therefore assigned the same tumor growth rate to both cells by changing the values of the *Γ_0_* and *α* parameters (see Table 1) of ATAC-4 cells to those of the A431/H9 cells. Other parameters for the LMB-2 case were kept as given in Tables 2 and 3. The parameters for the SS1P case were not changed. Then, we recalculated the tumor volume profiles for both immunotoxin cases. The resulting tumor volume profiles for the two cases are remarkably similar (Fig. 4B), indicating that these two immunotoxins are equally effective.

### The effect of antigen shedding depends on the number of antigen molecules on the cell surface and the endocytosis rate

Although the models predict a similar anti-tumor activity for SS1P and LMB-2, there are large differences in some of the parameter values. As summarized in Table 3, in comparison with SS1P targeting mesothelin, there are a smaller number of target antigen molecules (R* = 2×10^5^) for LMB-2, with a negligible shedding rate (*k_s_* = 0.002 hr^−1^) and which undergoes a slower endocytosis (*k_e_* = 0.08 hr^−1^ vs. 0.22 hr^−1^ for mesothelin). Also, the fraction of toxin translocated into the cytosol (*Ω*) was lower for LMB-2, which indicates that more toxin molecules are degraded in the endosome for LMB-2 than for SS1P. (See Table 1 for the names of the parameters.)

**Figure 5:**
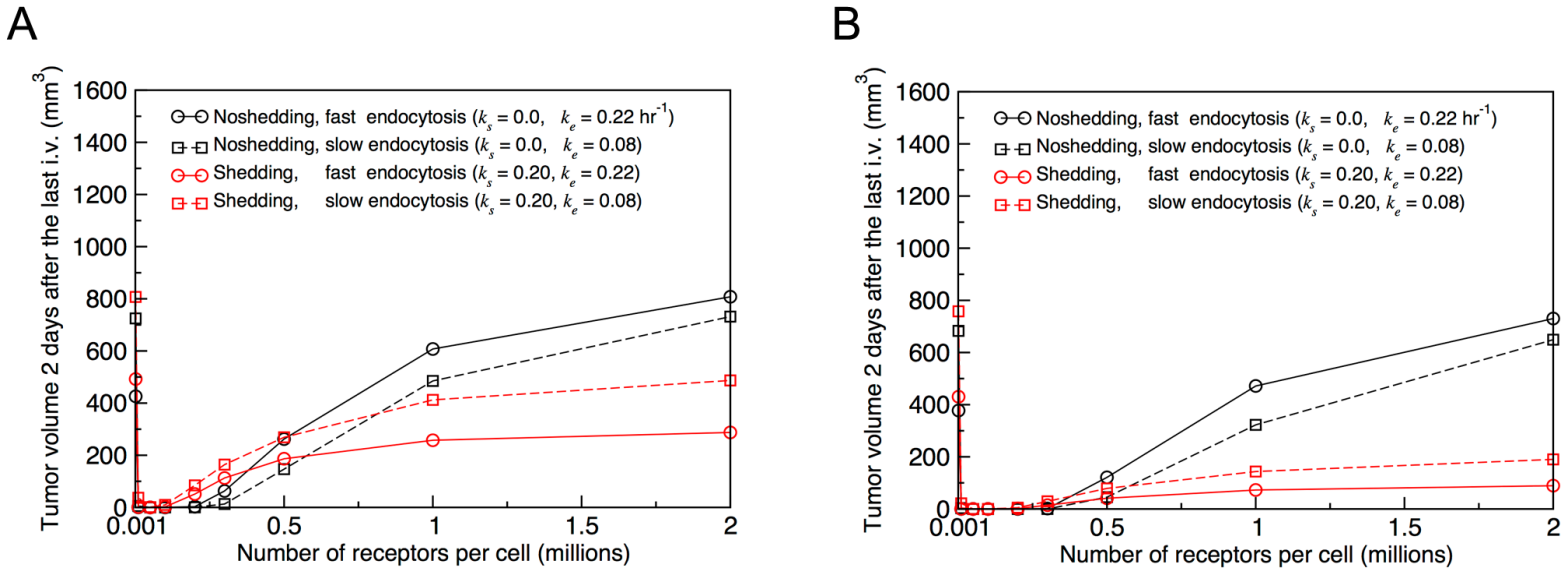
Predicted tumor volumes at two days after the last injection versus number of surface antigen molecules per cell (R*) for the A431/H9 cell tumor with 3×62 nM (A) or 3×124 nM (B) injections of SS1P for the shedding and no shedding cases with two different endocytosis rate constants (*k_e_* = 0.08 and 0.22 hr^−1^).

In an attempt to explain the similar efficacy of LMB-2 and SS1P and to understand these differences in parameter values between the two cases, we calculated the tumor volume profile for SS1P for R* values ranging from 10^3^ to 10^7^ for both shedding (*k_s_* = 0.2 hr^−1^) and non-shedding (*k_s_* = 0.0) cases, each using two different endocytosis rate constants. (All other parameters remain the same as those listed in Tables 2 and 3 for SS1P). The results are shown in Fig. 5, where the simulated tumor volumes 2 days after the third injection (day 12) of 62 nM (panel A) or 124 nM (panel B) are plotted at different R* values for both shedding and non-shedding cases of SS1P. The Figures show that there exists an optimal range of R* values where the immunotoxins are maximally effective. The optimal range varies depending on the dose applied: 10^4^<R*<10^5^ when 62 nM is given (Fig. 5A) and 10^4^<R*<2×10^5^ when 128 nM is given (Fig. 5B). For both shedding and non-shedding cases, immunotoxin becomes less effective as R* increases beyond these ranges, presumably because the binding site barrier increases.

One can also note that, for R* values of half a million or more, the receptor shedding is clearly beneficial for the immunotoxin delivery and its beneficial effect becomes more pronounced with increasing R*. (Compare the solid black and red lines in Figs. 5.) For smaller values of R*, however, shedding in fact reduces the effectiveness of immunotoxin. Thus, the beneficial effect of antigen shedding which we reported in the earlier study is more likely limited to the case of a large number of target binding sites on the cell when the binding site barrier effect is high.

Endocytosis rate also matters. Figs. 5 show that a smaller *k_e_* value makes immunotoxins more effective for the non-shedding case (black broken line), but less effective for the shedding case (red broken line). This finding can be explained along the following line of reasoning. In the non-shedding case, binding site barrier effect is the controlling factor as the tumor cells near the capillary act as a sink by absorbing a large amount of immunotoxin, preventing penetration and uniform distribution of immunotoxin into tumor tissues. A lower endocytosis rate helps in this case by reducing the immunotoxin loss to the sink thereby making more immunotoxin available for deeper penetration into the tumor tissue. In the shedding case, binding site barrier effect is not as important since the presence of free immunotoxin-receptor complex effectively circumvents the barrier. A larger *k_e_* value helps in this case because the antigen shedding competes with the endocytosis process to retard the internalization of immunotoxins.

Comparing Figs. 5A and 5B, we also note that increasing the dose level has a large effect when antigen is shed (red lines) but has relatively little effect when there is no shedding (black lines). This latter feature is the expected behavior when the binding site barrier is the limiting factor.

### The model predicts faster translocation rate for SS1P than LMB-2

Table 3 shows that the value of the *Ω* parameter is smaller for the LMB-2 than for the SS1P case, which implies that LMB-2 is degraded more in endosome (or less translocated to the cytosol) than SS1P. This is an expected result because, in terms of delivery efficiency, LMB-2 with a smaller receptor number (R* = 2×10^5^) and a negligible shedding (or non-shedding; *k_s_* = 0.0) should be better than SS1P with a larger receptor number (R* = 1×10^6^) and a substantial shedding (*k_s_* = 0.2 hr^−1^) (Fig. 5). Therefore, within our model, the only way the efficacies of SS1P and LMB-2 become similar when the natural growth rates of the different cell lines are made the same (Fig. 4B) is that LMB-2 is degraded more in endosome (or less translocated to the cytosol). This conclusion has some experimental support: the measured IC50 value for LMB-2 on ATAC-4 cells is 0.4 pM [25] whereas that for SS1P against A431/H9 cells is 0.2 pM (unpublished data). Although definitive conclusions cannot be made from these IC50 values, which depend sensitively upon details of the experimental conditions, they are consistent with the possibility that LMB-2 is less toxic on ATAC-4 cells than SS1P is on A431/H9 cells. We assume that this is possible if CD25 and mesothelin end up in different endosomal compartments upon endocytosis.

## Discussion

In our earlier study [5], we presented a mathematical model, which tracks immunotoxin concentrations in different compartments of a solid tumor tissue. For the first time, this model incorporated effects of antigen shedding into the detailed kinetic events that immunotoxin molecules encounter during their transit from the blood to tumor cells. We showed that this model reproduced dose-dependent antitumor activity of SS1P on the A431/H9 cell-derived tumor growing in mice. The model predicted that antigen shedding improved delivery efficiency of the immunotoxin in solid tumors.

In the present study, we improved the model by incorporating several new features and applied the improved model on two systems, the immunotoxin LMB-2 targeting CD25 of the ATAC-4 cell tumor as well as the original SS1P targeting mesothelin of the A431/H9 cell tumor.

One of these improvements is the introduction of a tumor volume dependent vascular permeability to better fit the experimental shed antigen level. In the absence of lymphatic drainage, the concentration of shed antigen in the extra-cellular space increases by shedding and decreases mainly by back-permeation. The shedding rate depends on the number of antigen molecules per cell and the number of cells per unit volume of the extra-cellular space. When immunotoxin is not given, both of these are constants, independent of the tumor size in our model. The back-permeation rate depends on the blood vessel surface area per volume of the extra-cellular space, which is also constant. Therefore, the steady state extra-cellular space concentration of shed antigen cannot depend on the tumor size in our model. However, the recent experiments on the mesothelin-expressing tumor [8] showed that, in the absence of immunotoxin, shed antigen level in the extra-cellular space increased with tumor size. This implies that the tumor capillary does not grow proportionally with the tumor volume as required by our model. Within the framework of our model, a simple way to produce a similar effect is to make the blood vessel less permeable as the tumor size increases. Clearly, this new scheme led to much better fits to the experimental shed antigen level (Fig. 2).

Another major modification is to allow all species, including immunotoxin and the shed antigen-immunotoxin complexes, to back-permeate from the extra-cellular space to the blood. In the previous model, we assumed that only the shed antigen could back-permeate. In a normal tissue, materials extravasate into the interstitial space mainly by the blood-tissue hydrostatic pressure differential [26] and are cleared from there by the lymphatic system; there should be little back permeation into the blood stream. In the tumor tissue, back permeation should increase both because there is no lymphatic drainage and because of the hydrostatic pressure that builds up in the interior of the tumor tissue [13]. The rate of back-permeation is not known, but presumably it approaches, but probably not exceeds, the forward permeation rate. In this study, we determined the back-permeation rate constant for the shed antigen by assuming that it is the same for both mesothelin and CD-25 and by using their blood and extra-cellular space concentrations in the absence of immunotoxin (see Methods and Text S1). We then assumed that the forward as well as the back-permeation rate constants for all molecular species were the same as the back-permeation rate constant determined for the free shed antigen. This probably over-estimates the back-permeation of the shed antigen-immunotoxin complex relative to that of the free shed antigen, since the complex molecule is larger in size, although permeability is not too sensitive to the molecular size in the 70 kD range [12], [27], and relative to the forward permeation of immunotoxin, in contrast to the under-estimation made in the previous model by dis-allowing the back-permeation altogether. Under this condition of relatively high back-permeation of the shed antigen-immunotoxin complex, the benefit of shedding is greatly reduced but still present in the case of SS1P (Fig. 4A).

This study also shows the value of considering more than one system. Whereas shedding is clearly beneficial for SS1P (compare red vs. black lines at R* value of 1 million in Fig. 5), it would reduce the efficacy of LMB-2 (compare red vs. black lines at R* value of 0.2 million in Fig. 5). When the number of target antigen molecules is large, as in the case of mesothelin-expressing A431/H9 cells, the free immunotoxin penetration is severely retarded by a significant binding site barrier and shedding is beneficial as the shed antigen acts as a protective carrier of immunotoxin, thus circumventing the binding site barrier effect. This finding confirms the previous result on the A431/H9/SS1P [5]. On the other hand, when the number of target antigen molecules is small, as in the case of CD25-expressing ATAC-4 cells, the binding site barrier is lower and the transport of the free immunotoxin in the extra-cellular space is less hindered. In this situation, the negative effects of antigen shedding become dominant: The shed antigen acts as a decoy to decrease the free immunotoxin level in the extra-cellular space and the antigen-immunotoxin complex can be shed, thus reducing the rate of productive entry of immunotoxin into the cell by endocytosis. This is in line with the other view that was suggested before [7], [11]. Therefore, our expanded perspective on the antigen shedding is that the shed antigen can act as either a promoter or a suppressor of immunotoxin deliveries depending on the number of target binding sites per cell.

In the previous work [5], we suggested that binding site barrier effect must exist also for natural extra-cellular signaling molecules operating in normal tissues and that receptor shedding could be a natural strategy to improve the range and duration of signaling molecules. The findings in this study now suggest that the effect of shedding can be either positive or negative depending on the number of receptors and that receptor shedding can be used to control the efficiency of signaling. However, the point at which the effect switches from being negative to positive will generally not be the same as that found in this study with the immunotoxins on cancer tissues. The switching point will depend on a number of factors including the endocytosis rate (see below), number of ligand molecules required to cause an effect in each cell, and the concentration of ligands.

The rate of entry of immunotoxin molecules into the cell is governed by several competing processes: the association with and dissociation from the surface-bound antigen, antigen shedding, and endocytosis. The two immunotoxins have a comparable association and dissociation rates. But the endocytosis rates are very different (Table 3). We find that a larger endocytosis rate does not always ensure an increased efficacy of immunotoxins. Our model shows (Fig. 5) that a smaller *k_e_* value is more effective for the non-shedding case where the binding site barrier is the main obstacle whereas a larger *k_e_* value is more effective for the shedding case. Also, except for the case of extremely small number of antigen molecules (<1000), in which the internalization of immunotoxin is severely restricted, a smaller number of binding sites is always better for both shedding and non-shedding cases. This last observation is supported by other models (see, for example, [28]) and serves as a warning that many cancer antigens, which are identified because of their overexpression in cancer cells, may not be the ideal targets for immunotherapy [29].

Since the number of binding sites, the shedding rate, and the endocytosis rate all favor LMB-2 over SS1P, LMB-2 should reach more cancer cells. However, the models that best fit the experimental data indicate that LMB-2 and SS1P have remarkably similar potencies (Fig. 4B). Within our model, these facts imply that the translocation efficiency (*Ω*) in endosome must be reduced significantly for LMB-2 (Table 3). Thus, rather surprisingly, our delivery simulation model suggests that CD25 and mesothelin go to different endosomal compartments where the cargo that they carried in is degraded or translocated into the cytosol at different rates. Obviously, any prediction made using a highly simplified mathematical model needs to be verified experimentally, but the fact that such a prediction can be made at all shows the usefulness of a comprehensive model.

## Materials and Methods

### Model system

A tumor is modeled as a collection of *m* identical representative units (RUs, Fig. 1). Each RU is a cylinder of 50 µm radius and has a cylindrical blood vessel at its center. The blood vessel is the source of immunotoxin. As the tumor grows or shrinks, the total number of RUs increases or decreases and the tumor volume is simply given by *m* times the volume of an RU.

**Figure 6:**
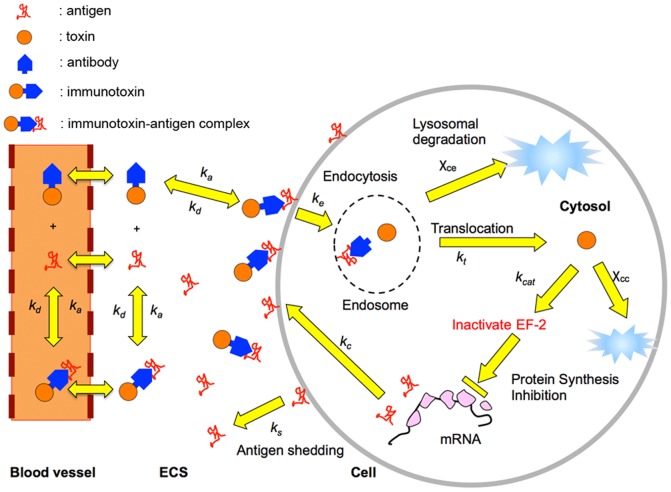
Kinetic events involved in immunotoxin-antigen binding and intracellular trafficking. Each yellow arrow indicates a kinetic step of the model. The tumor cell sheds the surface antigen and the surface complexed antigen at a certain rate. The immunotoxin molecule exiting from the capillary diffuses in the extra-cellular space and binds to either the surface antigen or shed antigen by the association reaction between the antigen and immunotoxin. The surface-bound immunotoxin is internalized by the receptor-mediated endocytosis and mostly inactivated in the endosomal stage. The surviving toxin translocates to the cytosol, where the toxin inhibits protein synthesis and eventually causes cell death. In non-intoxicated cells, the antigen is replenished by fresh protein synthesis.

**Figure 7:**
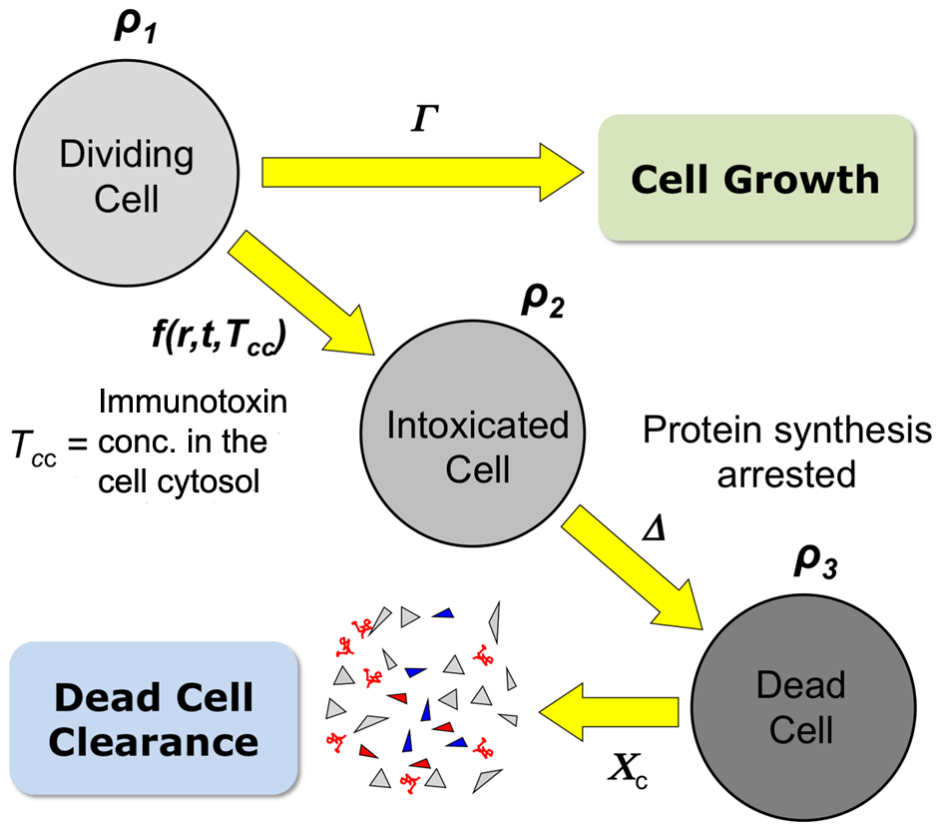
Kinetics of tumor cell population changes. The model defines three different tumor cell types: un-intoxicated, intoxicated, and dead cells, with densities *ρ_1_*, *ρ_2_*, and *ρ_3_*, respectively. The un-intoxicated cells proliferate, but the intoxicated and dead cells do not. The conversion from un-intoxicated to dead cells is irreversible. The number of the surface antigen molecules on an un-intoxicated cell remains constant, but it decays on an intoxicated cell through endocytosis and shedding without supply of newly synthesized internal antigen. Both protein synthesis and endocytosis have stopped in the dead cell, but the shedding and the on and off reaction between the surface antigen and immunotoxin still go on. The dead cells are removed from the tissue, presumably by macrophages, at a certain rate.

The model consists mainly of two sets of partial differential equations (see Text S1 for detailed equations). One set of equations governs immunotoxin concentrations in the blood, in the extra-cellular space (ECS), and in the three compartments of the tumor cell: surface, endosome, and cytosol (Fig. 6). The other set of differential equations describes density changes of three different types of tumor cells (Fig. 7): un-intoxicated (type 1), intoxicated (type 2) and dead (type 3) cells. The un-intoxicated cells divide and shed their surface antigens into the ECS. They become intoxicated by the action of immunotoxin. Protein synthesis is arrested in intoxicated cells, which do not produce new antigen molecules and do not divide, but endocytosis, intracellular trafficking, and surface antigen shedding are still presumed to go on until the cells die. Only the antigen shedding and the on and off reactions of free immunotoxins with the surface antigens are still presumed to go on in the dead cells, which nonetheless occupy volume in the tumor mass until physically cleared. Tumor cells in each RU can move out of RU as the tumor cells increase in number by cell division or into RU as space is created when intoxicated cells die and get cleared. A simple flux consideration of the cell flow in and out of the RU gives the governing equation for tumor volume profile with time (Eq. 8 in Text S1).

### Modifications and improvements

The basic concept and equations of the present model are the same as those of the earlier model [5], (See Text S1 for details) except for the modifications summarized below.

(i) The back-permeation into the blood vessel is allowed for all molecular species in ECS, including the shed antigen-immunotoxin complex, which was not allowed to back-permeate in the previous model. Specifically, for any relevant quantity *Q_i_* in ECS, the boundary condition at the blood vessel wall (*r_b_*) was given by allowing both forward and backward permeations across the blood vessel boundary:
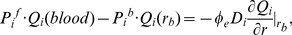
(1)where *P_i_^f^* and *P_i_^b^* are the forward (from blood to ECS) and backward (from ECS to blood) permeabilities of molecular species *i* (in unit of cm/s), *D_i_* is the diffusion constant of species *i* in ECS, and *φ_e_* is the volume ratio of the ECS to the RU.

(ii) The permeability of shed antigens and other relevant molecular species traveling across the blood vessel wall decreases when tumor volume becomes large. This makes the shed antigen level to increase with tumor volume (see Fig. 2) in line with the recent experimental observation [8] that the concentration of shed antigen is higher both in the ECS and in the blood when tumor size is large. In the previous model, the permeability was assumed to be constant, which resulted in a constant level of shed antigen concentration in the ECS, independent of the tumor size. In the current model, we introduce a permeability that varies from low *P_low_* to high *P_high_* values with tumor volume *V* according to
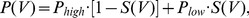
(2)where the sigmoidal switch function *S(V)* is given by
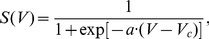
(3)where *a* (slope) and *V_c_* (center) are adjustable parameters. As explained in the Introduction and Discussion sections, this is an artificial way by which the model effectively accounts for the fact that the vasculature in large tumors is very aberrant and there exist pockets of poor blood supply.

(iii) The tumor cell growth rate constant used in the previous model has been replaced with a growth rate function *Γ(t)*, which decreases as the tumor volume increases, 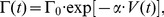
(4)where *V(t)* is the total tumor volume at time *t*, and *Γ_0_* and *α* are adjustable parameters. This is like Gompertz's model [30] except that it uses time for the exponential damping. We use tumor volume instead of time because our tumor volume can shrink (upon administration of immunotoxin) as well as grow with time. Use of Eq. (4) produces a noticeably better fit to the experimental tumor volume data at large tumor volumes both when immunotoxin is not given and long time after immunotoxin is given.

(iv) The cell intoxication follows the Michaelis-Menten type kinetics [31]. The cell intoxication rate is governed by the number of toxin molecules accumulated in the cell cytosol per tumor cell (*T_1_^CC^*). In the previous work, the use of a cell intoxication rate function of 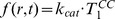 overestimated the rate at large *T_1_^CC^* and also produced a numerical instability when the type 1 cell density became small. In the current work, we use the new intoxication rate function
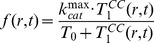
(5)where 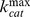, the maximum intoxication rate, and *T_0_*, the number of toxin molecules per cell at which the reaction rate is half of 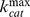, are adjustable parameters.

(v) The RU size has been expanded from 38 to 50 µm, so that the inter-capillary distance is 100 µm. The larger distance is more consistent with the median distance between cells to the blood vessel of, for example, 53–63 µm reported for the squamous cell carcinoma xenografts [32]. Also, cylindrical, rather than spherical, geometry is used for RU, with the cylinder radius set to 50 µm; the model is independent of the cylinder height, which never enters into the calculation. The blood vessel is also cylindrical. When a typical blood volume fraction of 5% is applied, the blood vessel radius (*r_b_*) becomes 11 µm. The cylindrical RU provides more layers of cells surrounding the blood vessel than a comparable RU with spherical geometry, but the model is rather insensitive to the geometry used: The quality of fit between the calculated and experimental tumor volume profiles is quite similar for SS1P whether cylindrical or spherical RU is used, but is slightly better with the cylindrical RU for LMB-2 (data not shown).

(vi) Finally, the cell density ρ* has been reduced to half of the value used before. (The value of ρ* given in the the earlier work (Table S1 in ref. [5]) has a typographical error – the actual value used in the model was 1.0_×_10^9^ cells/cm^3^ [25], not 1.9_×_10^9^ cells/cm^3^.) This is to effectively take into account the presence of non-tumor cells, e.g., stromal cells, macrophages, and other normal cells, which we assume to make up half of all the cells in the tumor tissue. Therefore, the cell density parameter in the current model refers to the three types (un-intoxicated, intoxicated, and dead) of tumor cells only. We kept the extra-cellular volume fraction *φ_e_* the same as before.

All the equations describing our model system are given in Text S1.

### Model parameters

All the parameters employed in the present work are listed in Table 1. Fig. S1 gives definitions of additional parameters used in the detailed model described in Text S1. Many parameter values in this model were adopted from the previous work [33] or published [7], [14], [15] or unpublished *in vivo* or *in vitro* experimental data. Other parameters were determined by the best fit to experimental data. For example, the endocytosis rate constant (*k_e_*) of SS1P was determined by fitting the *in vitro* dye-labeled SS1P internalization data on the A431/H9 tumor cell [7]. For LMB-2, the *k_e_* value was obtained by fitting *in vitro* internalization data of ^111^In-labeled LMB-2 into the ATAC-4 tumor cell [21]. The tumor growth rate parameters (*Γ_0_, α*) were determined by fitting to the experimental *in vivo* tumor growth curve (without immunotoxin injection). The shedding rate constant (*k_s_*), vascular permeability parameters (*P_low_, P_high_, V_c_*, and *a*), and the decay rate constant of the shed free antigen in the ECS and blood (*χ_efR_* and *χ_bfR_*) were obtained by fitting to the *in vivo* experimental shed antigen data in the ECS and blood [7], [8], [15]. Since we could not find reliable information on the permeation rates in the literature, we assumed that the forward and backward permeabilities of all molecular species were the same and equal to the back-permeability of shed antigens determined as above. Other remaining parameter values were determined by direct fits to experimental *in vivo* tumor volume profiles upon the administration of immunotoxins. Detailed procedures to obtain the model parameter values are given in Text S1. All the parameter values from our best fit with the current model are given in the Tables 2 and 3. Since the current model is modified in several different ways, the present parameter values for SS1P are not the same as before [5]. The model is variably sensitive to the model parameters. For a rather extensive sensitivity analysis of model parameters, see Chen et al. [33]. This analysis was done using an early model, but we expect the sensitivity to remain substantially the same for the new model, which is descended from and basically the same as the earlier model.

## Supporting Information

Figure S1 **Parameters involved in the kinetics of receptor recycling.** (Similar to Figure S2 of ref. 5 but redrawn.) The surface receptors (*R_s_*) are placed on the cell surface (yellow ribbon) with a rate constant *k_c_* and depleted by endocytosis (rate constant *k_e_*) and shedding (rate constant *k_s_*). The endocytosed receptors are mostly degraded (rate constant *k_deg_*). The remainder combines with the newly synthesized (at rate *G*) to form the internal receptor pool (*R_i_*) which is recycled to the surface (rate constant *k_c_*). For the type 1 cell, a steady state condition is imposed, such that the number of the surface receptors (*R_s_*) and the internal receptors (*R_i_*) per cell are kept constant. Furthermore, all endocytosed receptors are degraded and that all receptors that are presented on the surface are newly synthesized.
(PDF)

Text S1 **Full description of the mathematical model.**
(DOCX)
