# Supplementary figures and images for: Effect of Antigen Shedding on Targeted Delivery of Immunotoxins in Solid Tumors from a Mathematical Model
Source: PLoS One. 2014 Oct 24;9(10):e110716. doi: 10.1371/journal.pone.0110716 (PMC4208831; doi:10.1371/journal.pone.0110716)

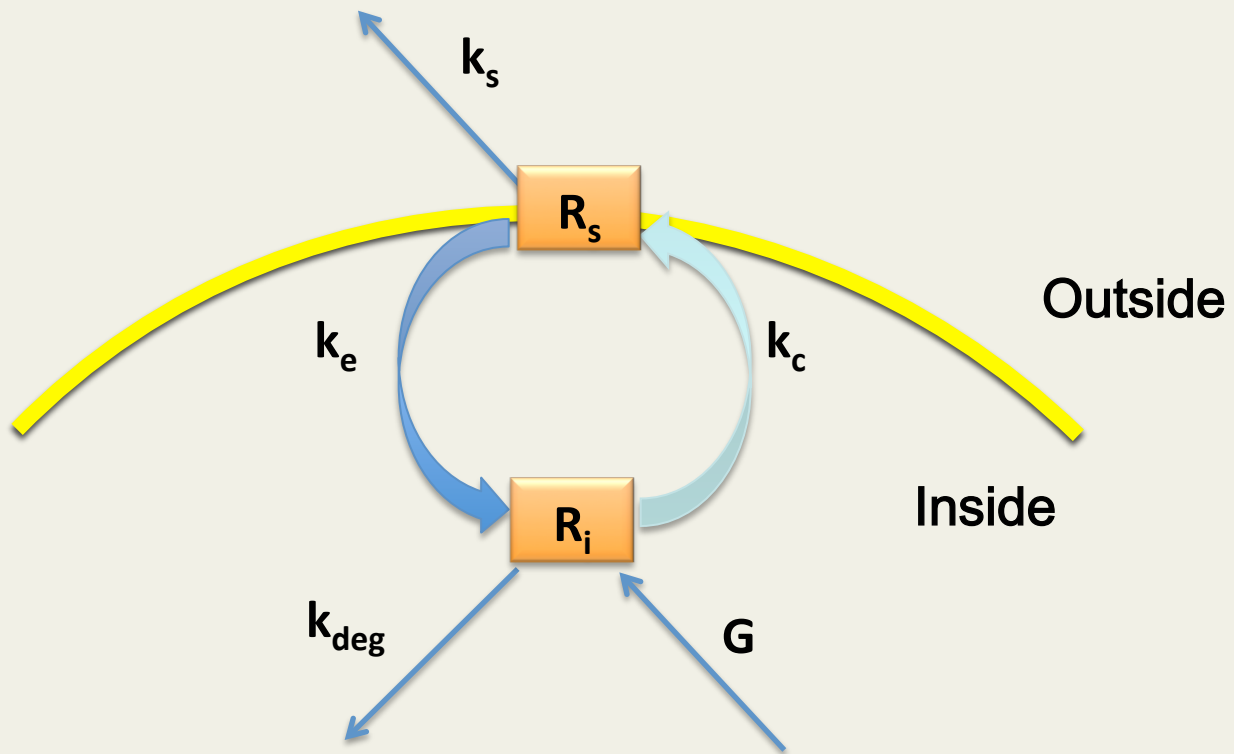

Supplement: Figure S1 — Parameters involved in the kinetics of receptor recycling. (Similar to Figure S2 of ref. 5 but redrawn.) The surface receptors (Rs) are placed on the cell surface (yellow ribbon) with a rate constant kc and depleted by endocytosis (rate constant ke) and shedding (rate constant ks). The endocytosed receptors are mostly degraded (rate constant kdeg). The remainder combines with the newly synthesized (at rate G) to form the internal receptor pool (Ri) which is recycled to the surface (rate constant kc). For the type 1 cell, a steady state condition is imposed, such that the number of the surface receptors (Rs) and the internal receptors (Ri) per cell are kept constant. Furthermore, all endocytosed receptors are degraded and that all receptors that are presented on the surface are newly synthesized. (PDF) [file pone.0110716.s001.pdf]
