# Supplementary material for: Effect of Antigen Shedding on Targeted Delivery of Immunotoxins in Solid Tumors from a Mathematical Model
Source: PLoS One. 2014 Oct 24;9(10):e110716. doi: 10.1371/journal.pone.0110716 (PMC4208831; doi:10.1371/journal.pone.0110716)
Supplement: Text S1 — Full description of the mathematical model. (DOCX) [file pone.0110716.s002.docx]

# Full Description of the Mathematical Model

## A. Model

### a. Components of tumor tissue

Our vascular tumor model consists of several different compartments:

**RU (representative unit):** This is a basic unit for describing the whole tumor tissue. We assume that the tumor tissue consists of *m* identical RUs. Each RU is a sphere (or a cylinder) of radius $r_{o}$ and a spherical (or a cylindrical) blood vessel of radius $r_{b}$ at its center. The volume of an RU is $\upsilon=\frac{4}{3}\pi r_{o}^{3}$ (spherical) or $\upsilon=\pi r_{o}^{2}h$ (cylindrical) where *h* is the cylinder height. The surface area of an RU is $\sigma_{0}=4\pi r_{o}^{2}$ (spherical) or $\sigma_{0}=2\pi r_{o}h$ (cylindrical). The value of the cylinder height, *h*, is not needed for the model since only the ratio between the surface area to volume is used in the model (see below).

**EVS (extra-vascular space):** Tumor tissue excluding the blood vessel region. EVS is mostly filled with cells, but about 10% is extra-cellular space (ECS). The EVS volume of one RU is $\upsilon'=\frac{4}{3}\pi(r_{o}^{3}-r_{b}^{3})$ (spherical) or $\upsilon'=\pi(r_{o}^{2}-r_{b}^{2})h$ (cylindrical). Lower case letters cs, ce, or cc label any quantity on the cell surface, in endosome, or in cytosol, respectively, expressed as concentration per EVS volume. Upper case letters, CS, CE, or CC labels the same quantity per cell.

**ECS (extra-cellular space):** Space unoccupied by cells in EVS. Here, .

**VS (vascular space):** Space of blood vessel. This constitutes 5% of RU. (). The surface area of VS for one RU is $\sigma_{b}=4\pi r_{b}^{2}$ (spherical) or $\sigma_{b}=2\pi r_{b}h$ (cylindrical).

### b. Tumor cell densities

In EVS, three different types of tumor cells are present: un-intoxicated (type 1), intoxicated (type 2), and dead (type 3) cells. The un-intoxicated cells can proliferate and shed the surface antigen molecules into ECS at a certain rate. When injected to the blood stream, the RITs in the blood enter the ECS and bind to the surface receptors on the un-intoxicated cell and are internalized and translocated to the cell cytosol, where the protein synthesis is inhibited by the catalytic action of the toxin (see Fig. 6 in the main text). The intoxicated cells are the ones in which the protein synthesis is terminated. Thus, their cell division is also terminated, but endocytosis, intra-cellular trafficking and surface antigen shedding still go on. The intoxicated cells eventually die. The dead cells do not show any cellular activity, but only shedding on the surface is allowed. The dead cells, which still occupy EVS, are cleared from it at certain rate. Upon the uptake of RITs by the un-intoxicated cells, the transition from un-intoxicated to dead cells via intoxicated cells is irreversible (see Fig. 7 in the main text). The densities of un-intoxicated ($\rho_{1}$), intoxicated ($\rho_{2}$), and dead tumor cells ($\rho_{3}$) in EVS are governed by

$\frac{\partial\rho_{3}}{\partial t}=U_{0}(\rho_{3})+\Delta\cdot\rho_{2}-X\cdot\rho_{3}$, (1)$\frac{\partial\rho_{2}}{\partial t}=U_{0}(\rho_{2})+f\cdot\rho_{1}-\Delta\cdot\rho_{2}$, (2)

$\frac{\partial\rho_{3}}{\partial t}=U_{0}(\rho_{3})+\Delta\cdot\rho_{2}-X\cdot\rho_{3}$, (3)

where

$U_{o}(x)\equiv-\frac{1}{r^{2}}\frac{\partial}{\partial r}\left(r^{2}u\cdot x\right)+\left[x(r_{o})-x(r_{b})\right]\frac{\sigma_{o}\cdot u(r_{o})}{\upsilon'}$ (spherical), (4a)

or

$U_{o}(x)\equiv-\frac{1}{r}\frac{\partial}{\partial r}\left(ru\cdot x\right)+\left[x(r_{o})-x(r_{b})\right]\frac{\sigma_{o}\cdot u(r_{o})}{\upsilon'}$ (cylindrical). (4b)

In Eqs. (1) - (3), *Γ*, *f*, $\rho^{*}$, and *Χ* are the tumor growth rate function, the intoxication rate function, cell death rate constant, and dead cell clearance rate constant, respectively. [Refer to Ref. [[1](#_ENREF_1)] for the detailed derivation of Eq. (4)]. The three different cell types in Eqs. (1) - (3) can migrate with a radial velocity *u(r)* and move in and out of the RU boundary. The cell migration velocity *u(r)* can be obtained at each time by imposing the condition that the total cell density $\rho^{*}$ is conserved. The governing equation for is

$\frac{\partial u}{\partial r}+\frac{2u}{r}=\frac{1}{\rho^{*}}(\Gamma\cdot\rho_{1}-X\cdot\rho_{3})$, (5)

where $\rho^{*}=\rho_{1}+\rho_{2}+\rho_{3}=const$. In the present work, the tumor growth rate function is newly defined as

$\Gamma(t)=\Gamma_{0}\cdot\exp[-\alpha\cdot V(t)]$, (6)

where *V(t)* is the total tumor volume at time *t*, and Γ_0_ and α are adjustable parameters, which are obtained from curve fits to experimental tumor growth profile without RIT injections. In the previous model[[1](#_ENREF_1)], α was set to zero. In general, the two-parameter expression for the tumor growth rate in Eq. (6) gives much improved fits to experimental tumor growth profiles in the absence of RITs. The intoxication rate function *f(r,t)* depends on the radial position and time since it also depends on the toxin concentration in the cytosol per type 1 cell i.e., $T_{1}^{CC}$. In the earlier work[[1](#_ENREF_1)], we employed$f(r,t)=k_{cat}\cdot T_{1}^{CC}$, but this function overestimated the rate at large $T_{1}^{CC}$ and also produced a numerical instability when the $\rho_{1}$density is very small. In the current work, to remedy these problems, we assume that the intoxication process by the cytosolic toxin follows the Michaelis-Menten type kinetics [[2](#_ENREF_2)]. The new intoxication rate function is

$f(r,t)=\frac{k_{cat}^{\max}\cdot T_{1}^{CC}(r,t)}{T_{0}+T_{1}^{CC}(r,t)}$, (7)

where $k_{cat}^{\max}$ is the maximum intoxication rate, and $T_{0}$ is the number of toxin molecules per type 1 cell at which the reaction rate is half of$k_{cat}^{\max}$. Note that $k_{cat}^{\max}$ and $T_{0}$ are adjustable parameters. The total tumor volume at time *t* can be obtained[[1](#_ENREF_1)] by considering the radial cell flow velocity across the RU boundary, $u(r_{0},t)$, using

$\frac{dV}{V}=\frac{\sigma_{0}\cdot u(r_{0})dt}{\upsilon'}$. (8)

### c. The amount of internalized toxin in EVS

In EVS, the number densities of the toxin on the cell surface, in the endosome, and cytosol of a cell of type *i* are denoted as $cR_{i}^{cs}$, $T_{i}^{ce}$, and $T_{i}^{cc}$, respectively. For the type 1 cells, the kinetic equations for the toxin concentrations in endosome and cytosol can be written as

$\frac{\partial T_{1}^{ce}}{\partial t}=U_{0}(T_{1}^{ce})+k_{e}\cdot cR_{1}^{cs}-k_{t}\cdot T_{1}^{ce}-\chi_{ce}\cdot T_{1}^{ce}-f\cdot T_{1}^{ce}$, (9a)

and

$\frac{\partial T_{1}^{cc}}{\partial t}=U_{0}(T_{1}^{cc})+k_{t}\cdot T_{1}^{ce}-\chi_{cc}\cdot T_{1}^{cc}-f\cdot T_{1}^{cc}$, (9b)

where $k_{e}$, $k_{t}$, $\chi_{ce}$, and $\chi_{cc}$are the rate constants of endocytosis, translocation from endosome into the cytosol, toxin inactivation in the endosome, and toxin inactivation in the cytosol, respectively. For the type 2 cells, the toxin concentrations are given by

$\frac{\partial T_{2}^{ce}}{\partial t}=U_{0}(T_{2}^{ce})+k_{e}\cdot cR_{2}^{cs}-k_{t}\cdot T_{2}^{ce}-\chi_{ce}\cdot T_{2}^{ce}+f\cdot T_{1}^{ce}-\Delta\cdot T_{2}^{ce}$, (10a)

and

$\frac{\partial T_{2}^{cc}}{\partial t}=U_{0}(T_{2}^{cc})+k_{t}\cdot T_{2}^{ce}-\chi_{cc}\cdot T_{2}^{cc}+f\cdot T_{1}^{cc}-\Delta\cdot T_{2}^{cc}$. (10b)

For the type 3 cells, the toxin concentration is given by

$\frac{\partial T_{3}^{c}}{\partial t}=U_{0}(T_{3}^{c})+\Delta\cdot T_{2}^{c}-X\cdot T_{3}^{c}$, (11)

where$T_{i}^{c}\equiv T_{i}^{ce}+T_{i}^{cc}$. Here, the cytosolic degradation in the dead cells was ignored, since all toxin contents in the dead cells are to be destroyed.

### d. Receptor concentrations in EVS

The number of surface receptors decreases as the receptors are internalized by endocytosis and also shed into ECS, but the surface receptors can be replenished by migration of newly synthesized receptors from the inside of cell. Previously, for type 1 and type 2 cells, ordinary differential equations for the total receptor number per cell on the cell surface ($tR_{i}^{CS}$) and inside the cell ($tR_{i}^{C}\equiv tR_{i}^{CE}+tR_{i}^{CC}$) were introduced as

$\frac{d(tR_{i}^{CS})}{dt}=-k_{e}\cdot tR_{i}^{CS}-k_{s}\cdot tR_{i}^{CS}+k_{c}\cdot tR_{i}^{C}$, (12a)

$\frac{d(tR_{i}^{C})}{dt}=k_{e}\cdot tR_{i}^{CS}-k_{\deg}\cdot tR_{i}^{C}-k_{c}\cdot tR_{i}^{C}+G_{i}$, (12b)

where $k_{s}$ is the shedding rate constant of the surface receptor, $k_{c}$ is the recycling rate (translocation from inside to the surface) constant of the internal receptors, $k_{\deg}$ is the degradation rate constant of the internalized receptors, and $G_{i}$ is the protein synthesis rate in cell type *i*. Note $G_{1}=G^{*}$ and$G_{2}=0$. For type 1 cells, we assume that the total numbers of surface and internalized receptors per cell are kept constant, such that$tR_{1}^{CS}=R^{*}$and$tR_{1}^{C}=\frac{k_{e}+k_{s}}{k_{c}}\cdot R^{*}$. For type 2 cells, the termination of protein synthesis makes the recycling of internal receptors to the cell surface insufficient to keep a constant surface receptor number. Thus, the total number of receptors on the type 2 cell surface decays with time.

In EVS, the number densities of total receptors on the cell surface ($tR_{i}^{cs}$) and those inside the cell ($tR_{i}^{c}$) are given by

$tR_{1}^{cs}=R^{*}\cdot\rho_{1}$, (13a)

$tR_{1}^{c}=\frac{k_{e}+k_{s}}{k_{c}}\cdot tR_{1}^{cs}=\frac{k_{e}+k_{s}}{k_{c}}\cdot R^{*}\cdot\rho_{1}$, (13b)

$\frac{\partial(tR_{2}^{cs})}{\partial t}=U_{0}(tR_{2}^{cs})-k_{e}\cdot tR_{2}^{cs}-k_{s}\cdot tR_{2}^{cs}+k_{c}\cdot tR_{1}^{c}+f\cdot tR_{1}^{cs}-\Delta\cdot tR_{2}^{cs}$, (14a)

$\frac{\partial(tR_{2}^{c})}{\partial t}=U_{0}(tR_{2}^{c})+k_{e}\cdot tR_{2}^{cs}-k_{c}\cdot tR_{2}^{c}-k_{\deg}\cdot tR_{2}^{c}+f\cdot tR_{1}^{c}-\Delta\cdot tR_{2}^{c}$, (14b)

$\frac{\partial(tR_{3}^{cs})}{\partial t}=U_{0}(tR_{3}^{cs})-k_{s}\cdot tR_{3}^{cs}+\Delta\cdot tR_{2}^{cs}-X\cdot tR_{3}^{cs}$, (15a)

$\frac{\partial(tR_{3}^{c})}{\partial t}=U_{0}(tR_{3}^{c})+\Delta\cdot tR_{2}^{c}-X\cdot tR_{3}^{c}$. (15b)

Following the receptor recycling kinetic scheme employed in the previous work (see Fig. S1), the values of$k_{\deg}$, $k_{c}$, and *G* an be simply obtained by

$k_{\deg}=k_{e}\cdot\frac{tR_{1}^{CS}}{tR_{1}^{C}}$, (16a)

$k_{c}=\frac{k_{e}+k_{s}}{k_{e}}\cdot k_{\deg}$, (16b)

$G=(k_{e}+k_{s})\cdot R^{*}$. (16c)

The values of$k_{e}$, $k_{s}$, and $R^{*}$ are usually available from experiment. Here we assume that $tR_{1}^{CS}/tR_{1}^{C}=100$ (The choice of this ratio does not make much difference in the simulation outcome).

### e. The mass balance equations in ECS

When RITs are injected to the blood stream, RITs in the blood stream enter the ECS via a permeation process. In ECS, RITs diffuse, undergo degradation processes, and bind to or dissociate from either surface bound antigens or shed antigens. We recognize four dynamic variables in ECS: free toxins (*T*), bound toxin on the cell surface (*scR*), shed free antigens (*efR*), and shed complexed antigens (*ecR*).

The governing equation for the concentration of free (uncomplexed) toxins, *T*, in ECS is

$\frac{\partial T}{\partial t}=U_{0}(T)+\frac{D_{efT}}{r^{2}}\frac{\partial}{\partial r}\left(r^{2}\frac{\partial T}{\partial r}\right)-\chi_{efT}\cdot T-k_{a}\cdot\sum_{i=1}^{3} sfR_{i}\cdot T+k_{d}\cdot\sum_{i=1}^{3} scR_{i}-k_{a}\cdot efR\cdot T+k_{d}\cdot ecR$,

(17)

where$D_{efT}$, $\chi_{efT}$, $k_{a}$, and $k_{d}$ are the diffusion constant of free RITs, the degradation rate constant of free toxin, the association rate constant of free toxin to the receptor, and the dissociation rate constant of the toxin-receptor complex, respectively. The mass balance equations for the toxin-receptor complex on the surface of three types of cells are

$\frac{\partial(scR_{1})}{\partial t}=U_{0}(scR_{1})-k_{e}\cdot scR_{1}-k_{s}\cdot scR_{1}+k_{a}\cdot sfR_{1}\cdot T-k_{d}\cdot scR_{1}-f\cdot scR_{1}$, (18a)

$\frac{\partial(scR_{2})}{\partial t}=U_{0}(scR_{2})-k_{e}\cdot scR_{2}-k_{s}\cdot scR_{2}+k_{a}\cdot sfR_{2}\cdot T-k_{d}\cdot scR_{2}+f\cdot scR_{1}-\Delta\cdot scR_{2}$,

(18b)

$\frac{\partial(scR_{3})}{\partial t}=U_{0}(scR_{3})-k_{s}\cdot scR_{3}+k_{a}\cdot sfR_{3}\cdot T-k_{d}\cdot scR_{3}+\Delta\cdot scR_{2}-X\cdot scR_{3}$. (18c)

The free receptor concentrations on the cell surface ($sfR_{i}$) are

for *i* =1, 3. (19)

The shed free receptor concentration $efR(r,t)$ in ECS is

$\begin{matrix} \frac{\partial(efR)}{\partial t}=U_{0}(efR)+\frac{D_{efR}}{r^{2}}\frac{\partial}{\partial r}\left[r^{2}\frac{\partial(efR)}{\partial r}\right]+ \\ k_{s}\cdot\sum_{i=1}^{3} sfR_{i}-k_{a}\cdot efR\cdot T+k_{d}\cdot ecR+\chi_{efT}\cdot ecR-\chi_{efR}\cdot efR \end{matrix}$, (20)

where $D_{efR}$ and $\chi_{efR}$ are the diffusion constant and the degradation rate constant of shed free antigens in ECS, respectively. The shed complexed receptor concentration *ecR(r,t)* in ECS is

$\frac{\partial(ecR)}{\partial t}=U_{0}(ecR)+\frac{D_{ecR}}{r^{2}}\frac{\partial}{\partial r}\left[r^{2}\frac{\partial(ecR)}{\partial r}\right]+k_{s}\cdot\sum_{i=1}^{3} scR_{i}+k_{a}\cdot efR\cdot T-k_{d}\cdot ecR-\chi_{ecR}\cdot ecR$,

(21)

where $D_{ecR}$ and $\chi_{ecR}$ are the diffusion constant and the degradation rate constant of shed complexed receptors in ECS.

In this work, for any relevant quantity $Q_{i}$ in ECS, the boundary condition (BC) at the blood vessel wall ($r_{b}$) was given by allowing both forward and backward permeations across the blood vessel boundary:

, (22)

where $P_{i}^{f}$ and $P_{i}^{b}$ are the forward (from blood to ECS) and backward (from ECS to blood) permeability functions of molecular species *i* in ECS (in unit of cm/s) and $D_{i}$ is the diffusion constant.

All the equations described above were given for the spherical geometry of RUs, but application to the cylindrical geometry is straightforward. For the cylindrical geometry of RUs, all the relevant mass balance equations are given by changing only the convection and diffusion terms to $-\frac{1}{r}\frac{\partial}{\partial r}\left(ru\right)$ and $\frac{D}{r}\frac{\partial}{\partial r}\left(r\frac{\partial}{\partial r}\right)$, respectively. Also, Eq. (5) is changed to$\frac{\partial u}{\partial r}+\frac{u}{r}=\frac{1}{\rho^{*}}(\Gamma\cdot\rho_{1}-X\cdot\rho_{3})$.

### f. The mass balance equations in blood

Generally, there are three different molecular species in the blood: free RIT, free antigen, and RIT-antigen complex. The free RIT level in blood ($T_{b}$) is

$\frac{dT_{b}}{dt}=m\left[P_{T}^{b}\cdot T(r_{b})-P_{T}^{f}\cdot T_{b}\right]\cdot\sigma_{b}/V_{b}-k_{a}\cdot bfR\cdot T_{b}+k_{d}\cdot bcR-\chi_{bfT}\cdot T_{b}$, (23)

where $P_{T}^{f}$ and $P_{T}^{b}$ are the forward and backward permeability functions for toxins at the blood vessel boundary, $\chi_{bfT}$ is the degradation rate constant of free toxins in blood, and *bfR* and *bcR* are the concentrations of shed free antigens and shed complexed antigens in blood, respectively. The shed antigen level in blood (*bfR*) is governed by

$\frac{d(bfR)}{dt}=m\left[P_{efR}^{b}\cdot efR(r_{b})-P_{efR}^{f}\cdot bfR\right]\cdot\sigma_{b}/V_{b}-k_{a}\cdot bfR\cdot T_{b}+k_{d}\cdot bcR-\chi_{bfR}\cdot bfR+\chi_{bfT}\cdot bcR$.

(24)

and the shed complexed antigen level in blood (*bcR*) by

$\frac{d(bcR)}{dt}=m\left[P_{ecR}^{b}\cdot ecR(r_{b})-P_{ecR}^{f}\cdot bcR\right]\cdot\sigma_{b}/V_{b}+k_{a}\cdot bfR\cdot T_{b}-k_{d}\cdot bcR-\chi_{bcR}\cdot bcR$. (25)

## B. Model parameters

Many parameter values in this model were taken from the previous work or published or unpublished experimental data. Remaining parameters were determined by direct fits to experimental shed antigen concentrations and tumor volume profiles upon injection of RITs. In order to reduce the number of parameters for fit, we set $P_{i}^{b}$ = $P_{i}^{f}$ with the same permeability value for all the molecular species *i* crossing the blood vessel wall. We also set the degradation rate constant in ECS equal for all molecular species, i.e., = =$\chi_{ecR}$. These simplifications enabled the fitting procedure more tractable without much affecting overall quality of fits.

### a. Pre-determined parameters

The diffusion constant of RITs in ECS (*D_efT_*) was taken from the earlier simulation study [[3](#_ENREF_3)]. Since the molecular weights of SS1P and LMB2 are almost the same, the same diffusion constant is assigned to both RITs. The ECS diffusion constants of shed free antigens *D_efR_* and RIT-antigen complexes *D_ecR_* were obtained by using an empirical relation *D∝(Mw)^-0.64^*, where *Mw* is molecular weight [[3](#_ENREF_3)]. For the parameters of the tumor growth rate function (*Γ*_0_, *α*), we acquired these values from an independent fit to each of the experimental tumor growth profiles available for the A431/H9 [[4](#_ENREF_4)] and ATAC-4 [[5](#_ENREF_5)] cells. The RIT translocation rate constant *k_t_* and RIT degradation rate constant in cytosol (*χ_cc_*) were fixed to the previously published values for both RITs [[1](#_ENREF_1)]. The RIT degradation rate constants in the blood phase *χ_bfT_* were obtained from the plasma half-lives for the first decaying phase (*t_α_*) experimentally measured for SS1P [[6](#_ENREF_6)] and LMB-2 [[7](#_ENREF_7)]. For the association rate constant of RIT to the receptor (*k_a_*) and the dissociation rate constant of SS1P from the receptor (*k_d_*), the experimental values previously determined by surface plasmon-resonance (unpublished) were employed, but for LMB-2, the values of the previous simulation study [[3](#_ENREF_3)] were used, since its experimental on and off values have not yet been available. For SS1P, the endocytosis rate constant *k_e_* was determined by fitting the *in vitro* dye-labeled SS1P internalization data on the A431/H9 tumor cell [[4](#_ENREF_4)]. For LMB-2, the *k_e_* value was also determined by fitting *in vitro* internalization data of ^111^In-labeled LMB2 into the ATAC-4 tumor cell [[8](#_ENREF_8)].

The *R** values for both A431/H9 and ATAC-4 tumor cells were experimentally known to be 1×10^6^ [[4](#_ENREF_4)] and 2×10^5^ [[9](#_ENREF_9)], respectively. In the earlier work, the tumor cell density of *ρ** **=**1.0×10^9^/cm^3^ was used [[10](#_ENREF_10)], but in the present work, this value is reduced by a factor of 2, by noting that tumor tissue also contains non-tumor cells with no more than 50% of total cellular composition.

### b. Determination of the values of the parameters *k_s_* and *P_efR_*

The shedding rate constant (*k_s_*) and the shed antigen permeability in both directions across the blood vessel wall ($P_{efR}^{f}$ and $P_{efR}^{b}$) can be determined by fitting to experimental shed antigen levels in ECS and in blood without toxin. When no RIT is present in the system, the shed antigen concentration in ECS, defined by Eq. (20), is given by

$\frac{\partial(efR)}{\partial t}=U_{0}(efR)+\frac{D_{efR}}{r^{2}}\frac{\partial}{\partial r}\left[r^{2}\frac{\partial(efR)}{\partial r}\right]+k_{s}\cdot sfR_{1}-\chi_{efR}\cdot efR$ (26)

and the boundary condition, Eq. (22).

The shed antigen level in blood in Eq. (24) is

$\frac{d(bfR)}{dt}=m\left[P_{efR}^{b}\cdot efR(r_{b})-P_{efR}^{f}\cdot bfR\right]\cdot\sigma_{b}/V_{b}-\chi_{bfR}\cdot bfR$, (27)

where *V_b_* is fixed to 5% of 20cc = 1cc for a mouse. As mentioned above, we set $P_{efR}^{f}$ = $P_{efR}^{b}$. An ECS-averaged value of the shed antigen level from Eq. (26) and the shed antigen level in blood from Eq. (27) were used to fit experimental shed antigen concentrations. Recent experimental data on SS1P [[4](#_ENREF_4)] indicated that the shed antigen levels have tumor volume dependence. As was the case of an earlier study [[1](#_ENREF_1)], using constant values of permeability did not yield reasonably acceptable fits to the experimental shed antigen data. In an attempt to produce a better fit, instead of using a fixed permeability value, we allowed that the permeability of shed antigens across the blood vessel boundary varies as tumor volume increases. One simple choice of including such volume dependence is to introduce a permeability function which varies from low $P_{low}$ to high $P_{high}$ values with tumor volume *V*, such that

$P(V)=P_{high}\cdot\left[1-S(V)\right]+P_{low}\cdot S(V)$, (28)

where the sigmoidal function *S(V)* is given by

$S(V)=\frac{1}{1+\exp\left[-a\cdot(V-V_{c})\right]}$. (29)

in which *a* (slope) and *V_c_* (center) are adjustable parameters. The parameters *k_s_*, *χ_efR_*, *P_low_*, *P_high_*, *a*, and *V_c_* can be determined from the fit to the experimental shed-antigen level in ECS. The *χ_bfR_* value (the shed antigen degradation rate constant in blood) can also be obtained by an additional fitting to the experimental shed antigen level in blood. Also, we required that SS1P and LMB-2 share a common set of permeability parameters *P_low_*, *P_high_*, *a*, and *V_c_*.

**c**. Determination of the remaining parameters from tumor volume fits.

Both ATAC-4 and A431/H9 cells were originated from the same cell line. Apparently, only difference is the expression of different antigens on their cell surface. Thus, the parameters *Δ* and *Χ* for these two target cells can be set equal. Furthermore, the parameters $k_{cat}^{\max}$ and *T_0_* for catalytic activities of cytosolic toxins can also be set the same for SS1P and LMB-2, since both RITs share an identical toxin part (Pseudomonas exotoxin).

Given pre-determined parameters and those parameters resulting from the shed antigen level fits, the remaining unknown parameters in our model were obtained by fitting to the experimental *in vivo* tumor volume profile upon the RIT injection (three injections every other day.) The initial guess for the parameter determination was taken from the previously published values [[1](#_ENREF_1)]. The detailed numerical simulation methods for solving the aforementioned mass balance equations were described in Ref. [[1](#_ENREF_1)].

## C. Approximate expressions for the concentration of shed antigen in the ECS and in the blood in the absence of immunotoxin.

### a. Shed receptor concentrations

(Shedding rate) = $m\cdot k_{s}\cdot R_{cs}\cdot\rho\cdot\upsilon^{EVS}$ (30)

where *m* = number of RUs in the tumor,

$k_{s}$ = shedding rate constant in units of 1/time,

$R_{cs}$ = number of receptors on the surface of one cell,

$\rho$ = density of cells in EVS of one RU,

$\upsilon^{EVS}$ = EVS volume of one RU.

(Shedding rate) = number of receptor molecules shed per unt time.

(Back permeation rate) = $m\cdot P\cdot A\cdot R_{cs}(r_{b})$ (31)

where *P* = back permeation rate constant in units of length/time,

*A* = capillary surface area per RU,

$R_{cs}(r_{b})$ = ECS concentration of shed antigen at the surface of capillary wall.

(Back permeation rate) = number of receptor molecules that cross the capillary wall from tumor to blood per unit time.

(Plasma clearance rate) = $\alpha\cdot R_{b}$ (32)

where α = plasma clearance rate constant in units of 1/time (called χ elsewhere),

*R_b_* = shed receptor concentration in the blood,

(Plasma clearance rate) = rate of decrease in receptor concentration in the blood with time.

### b. In the ECS

The ECS concentration of shed receptor is given by eqs. (30) and (31):

$\frac{dR_{ecs}\cdot\upsilon^{ECS}}{dt}=k_{s}\cdot R_{cs}\cdot\rho\cdot\upsilon^{EVS}-P\cdot A\cdot R_{cs}(r_{b})$, (33)

where *υ^ECS^* is the ECS volume of one RU.

Assuming that diffusion is infinitely fast (when diffusion is not fast, one must use the full mathematical model),

$R_{ecs}(r_{b})=R_{ecs}$, (34)

and that $R_{cs}$ is constant, eq. (33) can be solved to yield

$R_{ecs}=\frac{a}{b}(1-e^{-bt})$, (35)

where

$a=k_{s}\cdot R_{cs}\cdot\rho\cdot\upsilon^{EVS}/\upsilon^{ECS}$ (36)

and

$b=P\cdot A/\upsilon^{ECS}$. (37)

At long-time,

$R_{ecs}(\infty)=\frac{a}{b}=\frac{k_{s}\cdot R_{cs}\cdot\rho\cdot\upsilon^{EVS}}{P\cdot A}$, (38)

The same result is obtained by setting $\frac{dR_{ecs}}{dt}$ of eq. (33) to zero at long time. Thus, the steady state level of shed receptor in ECS is determined by the ratio of parameters, $\frac{k_{s}}{P}$.

### c. In the blood

The plasma concentration of shed receptor is given by eqs. (31) and (32):

$\frac{dR_{b}}{dt}=m\cdot P\cdot A\cdot R_{ecs}/\upsilon_{b}-\alpha\cdot R_{b}$. (39)

where *υ_b_* = volume of the blood.

Using eq. (35) for *R_ecs_* and assuming that *m* is constant (*m* increases with time, but we assume here that tumor growth is slow compared to the shedding and permeation processes; if not, one must use the full mathematical model), one can solve for *R_b_* to obtain

$R_{b}=c\left[\frac{1}{\alpha}(1-e^{-\alpha t})+\frac{1}{b-\alpha}(e^{-bt}-e^{-\alpha t})\right]$, (40)

where

$c=m\cdot k_{s}\cdot R_{cs}\cdot\rho\cdot\upsilon^{EVS}/\upsilon_{b}=a\frac{m\cdot\upsilon^{ECS}}{\upsilon_{b}}$. (41)

At long time,

$R_{b}(\infty)=\frac{c}{\alpha}=\frac{m\cdot k_{s}\cdot R_{cs}\cdot\rho}{\alpha}\cdot\frac{\upsilon^{EVS}}{\upsilon_{b}}$. (42)

The same result is obtained by setting $\frac{dR_{b}}{dt}$ of eq. (39) to zero at long time and replacing $R_{ecs}$ by $R_{ecs}(\infty)$ given in eq. (38). Thus, the steady state level of shed receptor concentration in the blood gives the value of *k_s_* and is independent of the value of *P*. Eq. (42) also shows that the blood level of shed antigen should depend linearly on tumor size.

From (38) and (42), the relation between $R_{ecs}(\infty)$ and $R_{b}(\infty)$ is

$R_{b}(\infty)=\frac{m\cdot P\cdot A}{\alpha\cdot\upsilon_{b}}\cdot R_{ecs}(\infty)$. (43)

## References

1. Pak Y, Zhang Y, Pastan I, Lee B (2012) Antigen shedding may improve efficiencies for delivery of antibody-based anticancer agents in solid tumors. Cancer research 72: 3143-3152.

2. Michaelis L, Menten ML (1913) The kenetics of the inversion effect. Biochemische Zeitschrift 49: 333-369.

3. Chen KC, Kim JH, Li XM, Lee B (2008) Modeling recombinant immunotoxin efficacies in solid tumors. Annals of Biomedical Engineering 36: 486-512.

4. Zhang Y, Hansen JK, Xiang L, Kawa S, Onda M, et al. (2010) A flow cytometry method to quantitate internalized immunotoxins shows that taxol synergistically increases cellular immunotoxins uptake. Cancer research 70: 1082-1089.

5. Singh R, Zhang Y, Pastan I, Kreitman RJ (2012) Synergistic antitumor activity of anti-CD25 recombinant immunotoxin LMB-2 with chemotherapy. Clinical cancer research : an official journal of the American Association for Cancer Research 18: 152-160.

6. Onda M, Nagata S, Tsutsumi Y, Vincent JJ, Wang Q, et al. (2001) Lowering the isoelectric point of the Fv portion of recombinant immunotoxins leads to decreased nonspecific animal toxicity without affecting antitumor activity. Cancer research 61: 5070-5077.

7. Tsutsumi Y, Onda M, Nagata S, Lee B, Kreitman RJ, et al. (2000) Site-specific chemical modification with polyethylene glycol of recombinant immunotoxin anti-Tac(Fv)-PE38 (LMB-2) improves antitumor activity and reduces animal toxicity and immunogenicity. Proceedings of the National Academy of Sciences of the United States of America 97: 8548-8553.

8. Kobayashi H, Kao CH, Kreitman RJ, Le N, Kim MK, et al. (2000) Pharmacokinetics of 111In- and 125I-labeled antiTac single-chain Fv recombinant immunotoxin. Journal of nuclear medicine : official publication, Society of Nuclear Medicine 41: 755-762.

9. Kreitman RJ, Bailon P, Chaudhary VK, FitzGerald DJ, Pastan I (1994) Recombinant immunotoxins containing anti-Tac(Fv) and derivatives of Pseudomonas exotoxin produce complete regression in mice of an interleukin-2 receptor-expressing human carcinoma. Blood 83: 426-434.

10. Kreitman RJ, Pastan I (1998) Accumulation of a recombinant immunotoxin in a tumor in vivo: fewer than 1000 molecules per cell are sufficient for complete responses. Cancer research 58: 968-975.
